# Benefit of continuous treatment for responders with newly diagnosed multiple myeloma in the randomized FIRST trial

**DOI:** 10.1038/leu.2017.111

**Published:** 2017-04-28

**Authors:** N J Bahlis, A Corso, L-O Mugge, Z-X Shen, P Desjardins, A-M Stoppa, O Decaux, T de Revel, M Granell, G Marit, H Nahi, H Demuynck, S-Y Huang, S Basu, T H Guthrie, A Ervin-Haynes, J Marek, G Chen, T Facon

**Affiliations:** 1 Tom Baker Cancer Center—University of Calgary, Calgary, Alberta, Canada; 2 Policlinico San Matteo Universita Di Pavia, Pavia, Italy; 3 Universitätsklinikum Jena Klinik für Innere Medizin II, Jena, Germany; 4 Ruijin Hospital, Shanghai Jiao Tong University, Shanghai, China; 5 Hôpital Charles LeMoyne, Longueuil, Canada; 6 Institut Paoli Calmettes, Marseille, France; 7 CHRU Hôpital Sud Médecine Interne, Rennes, France; 8 Hôpital d’Instruction des Armées PERCY, Paris, France; 9 Hospital de la Santa Creu i Sant Pau, Barcelona, Spain; 10 CHRU Hôpital du Haut Lévêque, CHU de Bordeaux, Pessac, France; 11 Karolinska University Hospital, Huddinge, Sweden; 12 H. Hart Ziekenhuis Roeselare-Menen, Roeselare, Belgium; 13 National Taiwan University Hospital, Taipei City, Taiwan; 14 New Cross Hospital, Wolverhampton, UK; 15 21st Century Oncology, Jacksonville, FL, USA; 16 Celgene Corporation, Summit, NJ, USA; 17 Service des Maladies du Sang, Hôpital Claude Huriez, CHRU Lille, Lille, France

## Abstract

The phase 3, randomized Frontline Investigation of Revlimid and Dexamethasone Versus Standard Thalidomide (FIRST) trial investigating lenalidomide plus low-dose dexamethasone until disease progression (Rd continuous) vs melphalan, prednisone and thalidomide for 12 cycles (MPT) and Rd for 18 cycles (Rd18) in transplant-ineligible patients with newly diagnosed multiple myeloma (NDMM) showed that Rd continuous prolonged progression-free survival and overall survival compared with MPT. A subanalysis of the FIRST trial was conducted to determine the benefits of Rd continuous in patients with NDMM based on depth of response. Patients randomized 1:1:1 to Rd continuous, Rd18 or MPT were divided into subgroups based on best response: complete response (CR; *n*=290), ⩾very good partial response (VGPR; *n*=679), ⩾partial response (PR; *n*=1 225) or ⩽stable disease (*n*=299). Over 13% of patients receiving Rd continuous who achieved ⩾VGPR as best response did so beyond 18 months of treatment. Rd continuous reduced the risk of progression or death by 67%, 51% and 35% vs MPT in patients with CR, ⩾VGPR and ⩾PR, respectively. Similarly, Rd continuous reduced the risk of progression or death by 61%, 54% and 38% vs Rd18 in patients with CR, ⩾VGPR and ⩾PR, respectively. In patients with CR, ⩾VGPR or ⩾PR, 4-year survival rates in the Rd continuous arm (81.1%, 73.1% or 64.6%, respectively) were higher vs MPT (70.8%, 59.8% or 57.2%, respectively) and similar vs Rd18 (76.5%, 67.7% and 62.5%, respectively). Rd continuous improved efficacy outcomes in all responding patients, including those with CR, compared with fixed duration treatment.

## Introduction

For patients with newly diagnosed multiple myeloma (NDMM), treatment objectives include disease control and extended survival.^1, 2^ Maintaining response is an important factor for prolonging survival in patients with multiple myeloma, in addition to achieving a high quality of response.^3, 4^ Moreover, patients with relapsed and refractory multiple myeloma are often more frail because of disease progression or comorbidities and may be unable to receive aggressive, yet efficacious, salvage therapies, highlighting the importance of maintaining first remission.^5^

Current standards of care for patients with NDMM who are ineligible for stem cell transplant are fixed duration combination therapy with melphalan, prednisone and thalidomide (MPT)^6, 7, 8^ or bortezomib, melphalan and prednisone in many parts of the world.^9, 10^ In addition, the pivotal phase 3, randomized international Frontline Investigation of Revlimid and Dexamethasone Versus Standard Thalidomide (FIRST) trial established lenalidomide plus dexamethasone (Rd) until disease progression (Rd continuous) as a new standard of care for this patient population.^11^ Specifically, data from the FIRST trial showed that Rd continuous significantly improved progression-free survival (PFS) compared with fixed duration MPT (12 cycles (72 weeks); hazard ratio (HR) for progression or death=0.72 (95% confidence interval (CI), 0.61–0.85); *P*<.001) or Rd for 18 cycles (72 weeks (Rd18); HR=0.70 (95% CI, 0.60–0.82); *P*<.001).^11^ At the interim overall survival (OS) analysis, Rd continuous also improved OS compared with MPT (HR for risk of death=0.78 (95% CI, 0.64–0.96)). Results from this trial led to the approval of lenalidomide plus low-dose dexamethasone for the treatment of previously untreated multiple myeloma in patients who were ineligible for transplant.^12, 13^

Results from a pooled analysis of the phase 3 MM-009 and MM-010 studies of lenalidomide plus low-dose dexamethasone in patients with relapsed and refractory multiple myeloma showed that patients who continued treatment experienced prolonged OS compared with those who discontinued treatment before disease progression.^14^ Furthermore, another pooled analysis of the same two trials found that higher quality responses to lenalidomide plus low-dose dexamethasone were associated with an OS benefit in patients with relapsed and refractory multiple myeloma.^15^ However, it is unknown whether continuing Rd therapy until disease progression results in improved outcomes for patients with NDMM in all response categories, especially for those who achieve complete remission. This retrospective *post hoc* analysis of the FIRST trial examined subgroups of patients based on best response achieved to determine the impact of continuing treatment with Rd on PFS and OS.

## Subjects and methods

### Study design

The phase 3, randomized, multicenter, open-label FIRST trial (MM-020) was conducted at 246 treatment centers in 18 countries in collaboration with the Intergroupe Francophone du Myélome (IFM 2007-01). Methods for this study have been previously described.^11^ Briefly, patients were randomized 1:1:1 by an interactive voice response system to Rd continuous until disease progression or unacceptable toxicity, Rd18 (72 weeks), or MPT for 12 cycles (72 weeks). Patients were stratified by age, country and International Staging System stage. In the Rd continuous and Rd18 arms, lenalidomide was administered 25 mg per day on days 1–21 and dexamethasone was given 40 mg on days 1, 8, 15 and 22 of a 28-day cycle. In the MPT arm, patients were administered melphalan 0.25 mg/kg on days 1–4, prednisone 2 mg/kg on days 1–4 and thalidomide 200 mg per day in 42-day cycles. Starting dose adjustments of dexamethasone, thalidomide and melphalan were made based on age, lenalidomide and melphalan based on level of renal function, and melphalan based on hematologic status. Thromboprophylaxis with low-dose aspirin, low-molecular-weight heparin, warfarin or equivalent was mandatory for all patients.

This study was approved by an institutional review board for each study site before initiation of any study procedures and was conducted in accordance with the Declaration of Helsinki and the principles of Good Clinical Practice (as outlined by the International Conference on Harmonisation E6 requirements). All patients provided informed consent. All authors had access to the primary clinical trial data and, with the sponsor, analyzed and interpreted the data. This trial is registered as NCT00689936.

### Patients

Eligible patients aged ⩾65 years or <65 years and ineligible for stem cell transplant had previously untreated, symptomatic and measurable MM as defined by International Myeloma Working Group criteria.^16^ Patients must have had an Eastern Cooperative Oncology Group performance status score of 0–2. Patients were excluded if they had received any prior antimyeloma treatment, with the exceptions of radiotherapy, bisphosphonates or short-term steroids. Patients with laboratory abnormalities including absolute neutrophil count <1.0 × 10^9^/l, untransfused platelet count <50 × 10^9^/l, or aspartate aminotransferase or alanine aminotransferase >3.0 × the upper limit of normal; renal failure requiring dialysis; peripheral neuropathy ⩾grade 2; or a history of malignancies, other than multiple myeloma, within the last 3 or fewer years were also excluded.

### Assessments

The primary endpoint was PFS, defined as time from randomization to the first documentation of disease progression or death because of any cause. Secondary endpoints included OS (time from randomization to death because of any cause), duration of response (time of initial response to confirmed disease progression), time to response (time from randomization to first response), time to second antimyeloma treatment (time from randomization to the first day the patient received second-line anitmyeloma treatment) and safety. Response and disease progression data reported here were investigator assessed using the International Myeloma Working Group criteria.^16^ An independent committee monitored efficacy and safety data throughout the study. Patients were retrospectively divided into four response subgroups: complete response (CR), ⩾very good partial response (VGPR; CR or VGPR), ⩾partial response (PR; CR, VGPR or PR) and ⩽stable disease (SD; SD or progressive disease). Patients who did not have any evaluable response were not included in this analysis. Landmark analyses of PFS and OS were conducted at 18 months after randomization for patients who achieved CR or ⩾VGPR. The primary comparators in the MM-020 trial, and hence this analysis, were Rd continuous vs MPT, as the study was not powered for the comparisons of Rd continuous vs Rd18 and Rd18 vs MPT. The purpose of this analysis was to evaluate the benefit of continuous Rd treatment vs MPT for patients with deep responses. As the response categories are mutually exclusive and add up to the total population, each individual response category (CR, VGPR and PR) should not be analyzed separately; therefore, responses were classified into three groups (CR, ⩾VGPR and ⩾PR).

### Statistical analysis

All response-evaluable patients were included in the efficacy analyses. Safety analyses included all response-evaluable patients who received at least 1 dose of study drug. The medians for time-to-event endpoints are based on Kaplan–Meier estimates, and the unstratified log-rank test was used for comparisons of these endpoints. The Cox proportional hazards model was used to estimate HRs and 95% CIs.

## Results

### Patients

Patients were grouped according to best response by investigator assessment using a data cutoff of 3 March 2014, with a median follow-up of 45.5 months. Of the 1623 patients in the intent-to-treat population, 1225 (75.5%) achieved ⩾PR, including 290 (17.9%) with CR and 679 (41.8%) with ⩾VGPR. There were 299 patients (18.4%) with a best response of ⩽SD (SD or progressive disease), including 266 (16.4%) with SD and 33 (2.0%) with progressive disease. An additional 99 patients (6.1%) were not evaluable for response and consequently were not included in this analysis. As indicated in the study flow diagram, 91 patients were still receiving treatment in the Rd continuous arm (see Supplementary Figure 1, available on the *Leukemia* website) and of these patients, 49 were in CR (43.0% of patients with CR), 80 with ⩾VGPR (31.0% of patients with ⩾VGPR) and 91 with ⩾PR (21.1% of patients with ⩾PR). There were no notable differences in baseline characteristics across response subgroups or treatment arms (Table 1).

### Responses, time to response and duration of response

**Figure 1:**
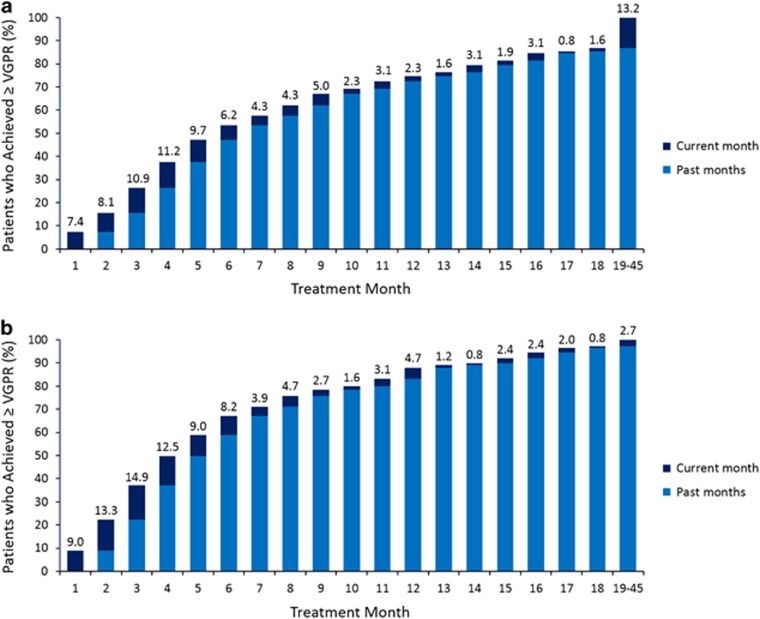
Cumulative response by treatment month for patients who achieved VGPR or better as best response. (**a**) Patients in the Rd continuous arm. (**b**) Patients in the Rd18 arm. Percentage shown is for patients achieving VGPR or better in the current month.

The overall response rate in the Rd continuous arm was 80.7% vs 67.3% and 78.6% in the MPT and Rd18 arms, respectively.^17^ For all responders, initial responses occurred in a median time of <2 months (1.0, 1.0 and 1.5 months for patients with CR; 1.1, 1.0 and 1.6 months for patients with ⩾VGPR; 1.8, 1.8 and 2.8 months for patients with ⩾PR; Supplementary Table 1) in the Rd continuous, Rd18 and MPT arms, respectively. Patients with high-quality response (that is, ⩾VGPR) as best response tended to have faster times to initial response across all treatment arms. Of patients who achieved CR, median time to first CR was 10.4, 8.3 and 11.3 months in the Rd continuous, Rd18 and MPT arms, respectively. Median time to first VGPR was 5.6, 4.1 and 6.2 months for patients who achieved VGPR in the Rd continuous, Rd18 and MPT arms, respectively. Of patients in the Rd continuous arm who achieved ⩾VGPR as best response, 88.8% (*n*=229) had a PR as initial response and 13.2% (*n*=34) achieved ⩾VGPR beyond 18 months of treatment (Figure 1a). In contrast, 2.7% (*n*=7) of patients who achieved ⩾VGPR as best response with Rd18 did so beyond 18 months of treatment (Figure 1b).

**Figure 2:**
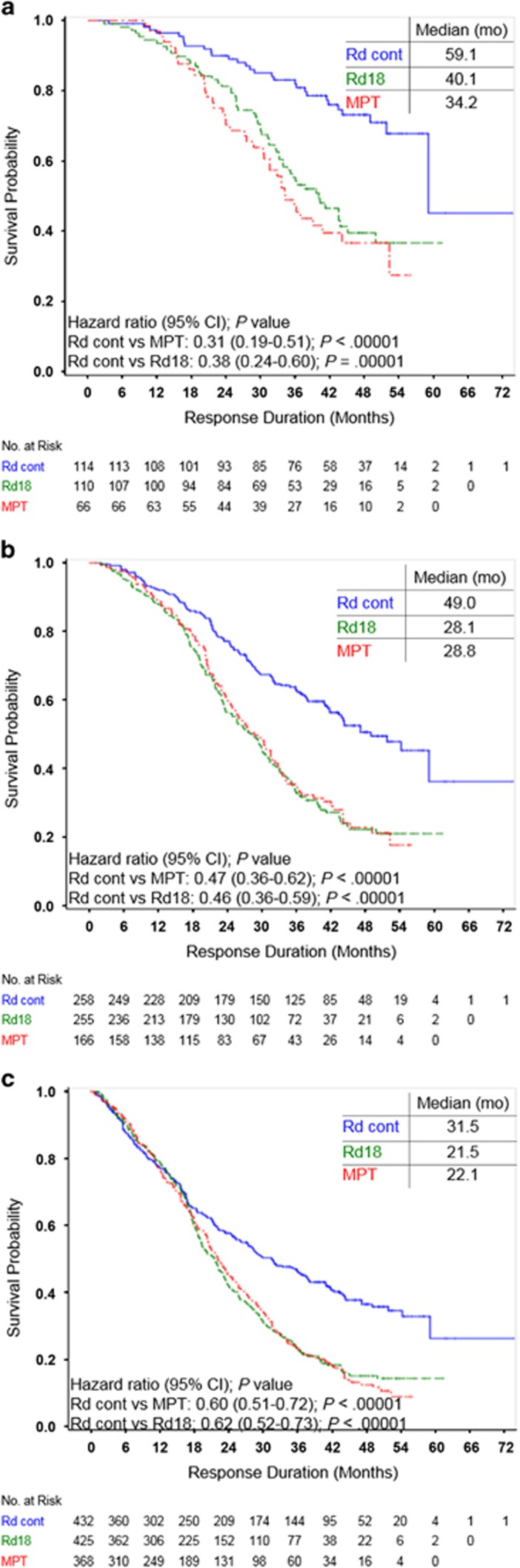
Kaplan–Meier estimates of duration of response. (**a**) Patients who achieved CR. (**b**) Patients who achieved VGPR or better. (**c**) Patients who achieved PR or better.

Duration of response was substantially longer with Rd continuous vs MPT and Rd18 across all response subgroups, especially in patients with ⩾VGPR and those with CR (Figure 2). In particular, in patients achieving CR, responses were sustained by an additional 19 months or more in patients receiving Rd continuous vs MPT or Rd18 (median duration of response, 59.1 vs 34.2 or 40.1 months, respectively). A similar magnitude of improvement with Rd continuous was also observed in patients achieving ⩾VGPR compared with MPT or Rd18. Responses were also maintained by an additional 9–10 months in all responders (⩾PR) who received Rd continuous treatment compared with either fixed duration regimen (MPT or Rd18). Subgroup analyses for duration of response in patients with ⩾VGPR are presented in Supplementary Figure 2.

### Progression-free survival

**Figure 3:**
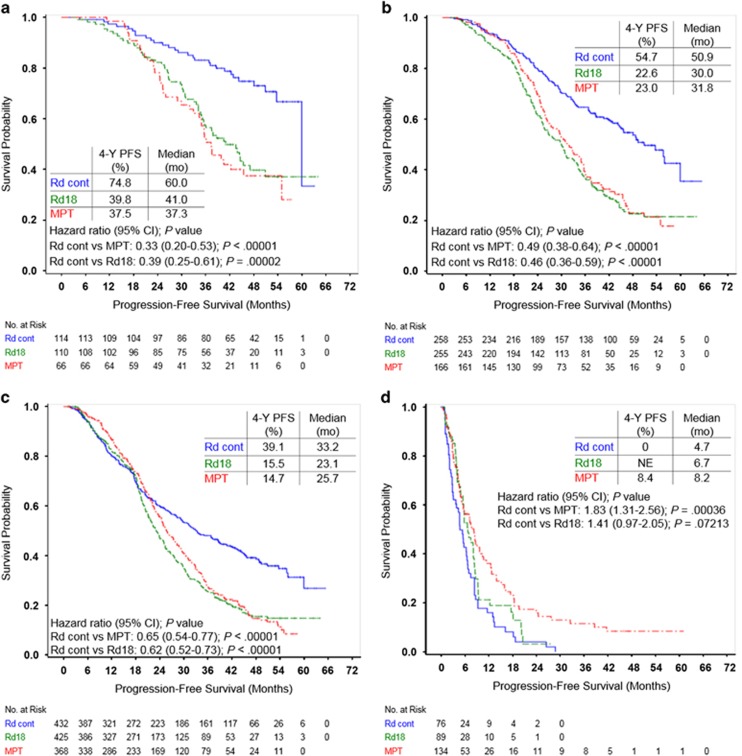
Kaplan–Meier estimates of PFS. (**a**) Patients who achieved CR. (**b**) Patients who achieved VGPR or better. (**c**) Patients who achieved PR or better. (**d**) Patients who achieved no better than SD. NE indicates not estimable.

Rd continuous treatment prolonged PFS in responding patients compared with MPT and Rd18, with a reduction in the risk of progression or death by 35% and 38%, respectively (Figure 3). This benefit was even more pronounced in patients with higher quality responses. Patients who achieved CR or ⩾VGPR had a 67% and 51% reduction in the risk of progression or death with Rd continuous vs MPT, respectively. Similarly, Rd continuous reduced the risk of progression or death by 61% and 54% vs Rd18 in patients with CR and ⩾VGPR, respectively. Rates of 4-year PFS were nearly doubled with Rd continuous vs MPT or Rd18 for those achieving CR (74.8% vs 37.5% or 39.8%, respectively) and more than doubled in patients achieving ⩾VGPR (54.7% vs 23.0% or 22.6%, respectively) or ⩾PR (39.1% vs 14.7% or 15.5%, respectively). A PFS benefit was observed with higher quality responses within all treatment arms (Supplementary Figure 3).

To further evaluate continuous treatment with Rd vs fixed duration treatment with MPT among patients with deep responses, landmark analyses of PFS were performed at 18 months for patients with CR or ⩾VGPR. Among the 259 patients who achieved CR and were evaluable at this time point, median PFS was 42.0 months with Rd continuous compared with 21.9 months with MPT (HR=0.29 (95% CI, 0.17–0.50); *P*<0.00001). At the 18-month landmark analysis of patients with ⩾VGPR (*n*=540), median PFS was 38.0 months with Rd continuous vs 17.1 months with MPT (HR=0.41 (95% CI, 0.30–0.56); *P*<0.00001).

### Overall survival

**Figure 4:**
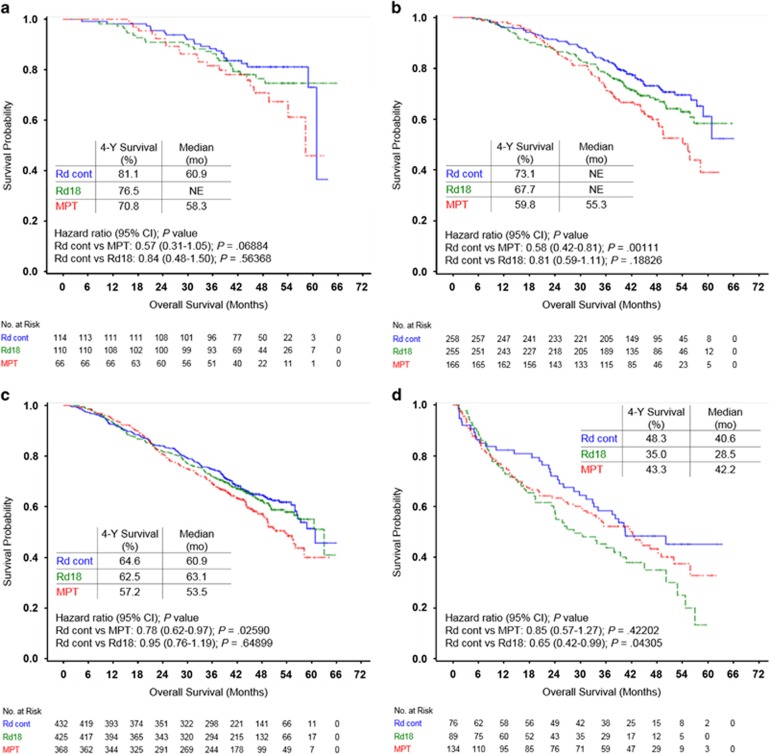
Kaplan–Meier estimates of OS. (**a**) Patients who achieved CR. (**b**) Patients who achieved VGPR or better. (**c**) Patients who achieved PR or better. (**d**) Patients who achieved no better than SD.

After a median follow-up of 45.5 months, Rd continuous improved survival in all subgroups of responders compared with MPT (Figure 4). Similar to PFS, patients with deeper responses also showed greater OS benefits with Rd continuous vs MPT, with reductions in the risk of death of 43%, 42% and 22% in patients with CR, ⩾VGPR and ⩾PR, respectively (Figure 4). Rates of 4-year survival of 81.1%, 73.1% and 64.6% were observed with Rd continuous compared with 70.8%, 59.8% and 57.2% with MPT in patients with CR, ⩾VGPR and ⩾PR, respectively. No OS benefits were observed in patients with ⩽SD. In the Rd18 arm, 4-year survival rates were 76.5%, 67.7% and 62.5% in patients with CR, ⩾VGPR and ⩾PR, respectively.

Landmark analyses were performed at 18 months evaluating OS of patients with CR or ⩾VGPR. Among the 276 patients who achieved CR and were evaluable at this time point, median OS was 42.9 months with Rd continuous compared with 40.3 months with MPT (HR=0.57 (95% CI, 0.30–1.08); *P*=0.082). At the 18-month landmark analysis of patients with ⩾VGPR (*n*=624), median OS was not reached with Rd continuous vs 37.3 months with MPT (HR=0.50 (95% CI, 0.35–0.72); *P*=0.00016).

### Time to second-line antimyeloma therapy

In line with results showing prolonged PFS, time to second-line antimyeloma treatment was considerably prolonged in patients treated with Rd continuous vs MPT or Rd18 across all subgroups of responding patients (Table 2). Median time to second-line antimyeloma treatment was extended by 18.5 (HR=0.28), 23.0 (HR=0.40) and 17.8 months (HR=0.64) with Rd continuous compared with MPT in patients achieving CR, ⩾VGPR and ⩾PR, respectively. Similarly, Rd continuous prolonged time to second-line antimyeloma therapy by 8.1 (HR=0.36), 20.8 (HR=0.45) and 18.0 months (HR=0.70) compared with Rd18 in patients achieving CR, ⩾VGPR and ⩾PR, respectively. Of note, the 258 patients who achieved ⩾VGPR in the Rd continuous arm had a median time to second-line antimyeloma therapy of approximately 5 years. Time to second-line antimyeloma treatment was similar across treatment arms in patients with ⩽SD.

### Safety

Grade 3/4 treatment-emergent adverse events of interest across response subgroups are presented in Table 3. The safety profile of each treatment was generally consistent across response subgroups. However, for patients in the Rd continuous and Rd18 arms, lower rates of grade 3/4 neutropenia and anemia were observed with a higher quality response.

Rates of dose reductions, dose interruptions and discontinuations of lenalidomide because of treatment-emergent adverse events were generally higher with Rd continuous vs fixed duration Rd18 treatment in patients with responses of CR, ⩾VGPR or ⩾PR. Rates of lenalidomide discontinuation with Rd continuous were generally comparable with rates of thalidomide discontinuation with fixed duration MPT across all response subgroups. In the 271 patients in the Rd continuous arm treated for ⩾18 months, 10.3% (*n*=28), 11.1% (*n*=30), 11.4% (*n*=31) and 13.3% (*n*=36) of patients were being treated with lenalidomide without dexamethasone at month 18, 24, 30 and 36, respectively. In these 271 patients, the median durations of lenalidomide and dexamethasone treatments were 36.5 and 29.8 months, respectively, and the median duration of lenalidomide treatment after dexamethasone discontinuation was 16.1 months. In addition, 15.1% (*n*=41), 10.3% (*n*=28), 8.5% (*n*=23), 5.9% (*n*=16) and 4.1% (*n*=11) of patients in the Rd continuous arm treated for ⩾18 months were receiving lenalidomide alone without dexamethasone for at least 12, 18, 24, 30 and 36 months, respectively. Of patients who remained on treatment in the Rd continuous arm at 1 year (*n*=422), 2 years (*n*=276) and 3 years (*n*=171), 28.0%, 38.8% and 50.3%, respectively, were not receiving lenalidomide at their initial dose within the previous six cycles of treatment.

## Discussion

This retrospective *post hoc* analysis of the FIRST trial, the largest prospective phase 3 study of transplant-ineligible patients with NDMM to date, clearly demonstrates the benefits attained by continuing treatment with Rd until disease progression in all responding patients, irrespective of the quality of response. Greater clinical benefits were seen with Rd continuous in patients with a higher quality of response, with larger differences in PFS and OS between Rd continuous and MPT observed in patients with CR. In all groups of responders (CR, ⩾VGPR and ⩾PR), the rate of 4-year PFS was approximately doubled or more with Rd continuous compared with MPT or Rd18, reaching almost 75% for patients with CR compared with 38% or 40%, respectively. An improvement in OS was also seen with Rd continuous for all groups of responders, with an increase observed in 4-year survival compared with MPT. Although the study was not powered for the comparison of Rd continuous vs Rd18, rates of 4-year survival were similar with Rd continuous vs Rd18 for all groups of responders. Notably, >13% of patients with a high-quality response achieved ⩾VGPR after 18 months of treatment with Rd, highlighting the importance of Rd continuous treatment.

Although it is known that greater depth of response is associated with improved outcomes in patients with multiple myeloma, it remains to be determined whether continuing therapy once complete or high-quality responses are achieved improves patient outcomes.^18, 19, 20, 21, 22^ This analysis of the FIRST trial demonstrates that Rd continuous compared with MPT or Rd18 resulted in significantly more durable responses and prolonged PFS in all response subgroups, including those with CR. Furthermore, these differences in PFS between Rd continuous and MPT were preserved in the 18-month landmark analyses of patients who achieved CR or ⩾VGPR. In the FIRST trial, Rd continuous treatment induced and maintained durable responses that were prolonged by 19 months or more in patients with CR or ⩾VGPR compared with MPT and Rd18. Importantly, the superior durability of high-quality responses, CR or ⩾VGPR, with Rd continuous compared with MPT resulted in a respective 10.3% and 13.3% improvement in 4-year survival.

This subanalysis of the FIRST trial is in agreement with the previous findings in favor of depth of response as a surrogate marker for survival, and also shows an added benefit of treatment duration. One retrospective analysis reported that approximately 40% of patients who had achieved CR had relapsed at the time of analysis, highlighting the need for long-term disease control to improve outcomes.^22^ Our results confirm that it is not only important to achieve a response, but that responses can be maintained by long-term treatment and, consequently, PFS can be further improved in patients with deep responses. This subanalysis demonstrates that continuous treatment significantly benefits all patients, regardless of best response. In fact, the rate of 4-year PFS with Rd continuous in all responders ⩾PR (39.1%) was comparable to that in patients with CR treated with MPT (37.5%) or Rd18 (39.8%). This highlights the importance of factors beyond quality of response for determining outcomes, such as maintaining a response with continuous treatment. Of note, 91 responding patients (⩾PR) were still on Rd continuous treatment at the time of data cutoff, including 49 patients with CR (which constituted nearly half the patients who achieved CR with Rd continuous), and 80 patients with ⩾VGPR. There were marginal survival benefits for responders with Rd continuous vs Rd18; interestingly, the benefits were more pronounced in patients with CR or ⩾VPGR.

With appropriate dose modifications, continuous treatment with Rd until disease progression was feasible, resulted in rapid and sustained disease control, and improved long-term survival outcomes vs treatment with MPT. These data support the use of Rd continuous therapy beyond achievement of best response until disease progression as a standard treatment regimen for transplant-ineligible patients with NDMM, especially patients who achieve high-quality responses.

## Figures and Tables

**Table 1:** Baseline patient characteristics by response subgroups

*Characteristic*	*CR (*n=*290)*			⩾*VGPR (*n=*679)*			⩾*PR (*n=*1225)*			⩽*SD (*n=*299)*		
	*Rd Cont (*n=*114)*	*Rd18 (*n=*110)*	*MPT (*n=*66)*	*Rd Cont (*n=*258)*	*Rd18 (*n=*255)*	*MPT (*n=*166)*	*Rd Cont (*n=*432)*	*Rd18 (*n=*425)*	*MPT (*n=*368)*	*Rd Cont (*n=*76)*	*Rd18 (*n=*89)*	*MPT (*n=*134)*
Median age (range), years	73.0 (48–89)	72.0 (40–87)	72.0 (51–90)	72.0 (44–91)	72.0 (40–89)	72.0 (51–90)	73.0 (44–91)	72.0 (40–89)	72.5 (51–91)	72.5 (61–88)	75.0 (55–85)	73.5 (53–92)
Age >75 years, *n* (%)	39 (34)	28 (25)	18 (27)	84 (33)	81 (32)	58 (35)	145 (34)	139 (33)	114 (31)	25 (33)	43 (48)	50 (37)
Male, *n* (%)	64 (56)	58 (53)	36 (55)	147 (57)	133 (52)	87 (52)	239 (55)	220 (52)	196 (53)	40 (53)	46 (52)	69 (51)
												
*ECOG PS*, n *(%)*												
0	38 (33)	32 (29)	17 (26)	84 (33)	77 (30)	46 (28)	130 (30)	135 (32)	109 (30)	21 (28)	21 (24)	38 (28)
1	52 (46)	55 (50)	32 (48)	122 (47)	119 (47)	89 (54)	214 (50)	205 (48)	190 (52)	34 (45)	45 (51)	68 (51)
2	24 (21)	23 (21)	16 (24)	51 (20)	59 (23)	29 (17)	86 (20)	85 (20)	67 (18)	20 (26)	23 (26)	27 (20)
⩾3	0	0	0	1 (0.4)	0	0	1 (<1)	0	0	0	0	0
Missing	0	0	1(2)	0	0	2 (1)	1 (<1)	0	2 (1)	1 (1)	0	1 (1)
												
*ISS stage*, n *(%)*												
I/II	78 (68)	69 (63)	43 (65)	160 (62)	155 (61)	91 (55)	263 (60.9)	273 (64.2)	232 (63.0)	49 (64.5)	36 (40.4)	71 (53.0)
III	36 (32)	41 (37)	23 (35)	98 (38)	100 (39)	75 (45)	169 (39.1)	152 (35.8)	136 (37.0)	27 (35.5)	53 (59.6)	63 (47.0)
												
*CrCl (ml/min)*, n *(%)*												
<30	7 (6)	12 (11)	7 (11)	14 (5)	22 (9)	18 (11)	29 (7)	31 (7)	30 (8)	7 (9)	11 (12)	19 (14)
⩾30 to <50	23 (20)	14 (13)	12 (18)	59 (23)	48 (19)	34 (20)	107 (25)	84 (20)	74 (20)	13 (17)	28 (31)	33 (25)
⩾50 to <80	59 (52)	57 (52)	18 (27)	121 (47)	119 (47)	63 (38)	193 (45)	205 (48)	157 (43)	36 (47)	35 (39)	51 (38)
⩾80	25 (22)	27 (25)	29 (44)	64 (25)	66 (26)	51 (31)	103 (24)	105 (25)	107 (29)	20 (26)	15 (17)	31 (23)
												
*Cytogenetics*, n *(%)*^a^												
High risk	5 (4)	7 (6)	0	13 (5)	18 (7)	5 (3)	33 (7)	35 (8)	32 (9)	7 (9)	12 (13)	13 (10)
Standard risk	42 (37)	45 (41)	35 (53)	101 (39)	99 (39)	80 (48)	166 (38)	167 (39)	146 (40)	30 (39)	33 (37)	42 (31)

Abbreviations: Cont, continuous; CR, complete response; CrCl, creatinine clearance; ECOG PS, Eastern Cooperative Oncology Group performance status; ISS, International Staging System; MPT, melphalan, prednisone and thalidomide; PR, partial response; SD, stable disease; VGPR, very good partial response.

a High risk includes del(17p), t(14;16) and t(4;14); standard risk includes all patients not categorized as high risk.

**Table 2:** Time to second-line antimyeloma treatment by response subgroups

	*CR (*n=*290)*	⩾*VGPR (*n=*679)*	⩾*PR (*n=*1225)*	⩽*SD (*n=*299)*
*Median time to second-line antimyeloma treatment, months*				
Rd continuous	60.7	60.7	49.8	6.9
Rd18	52.6	39.9	31.8	7.9
MPT	42.2	37.7	32.0	7.8
				
*HR (95% CI)*				
Rd continuous vs MPT	0.28 (0.16–0.49)	0.40 (0.30–0.55)	0.64 (0.53–0.78)	1.26 (0.91–1.74)
Rd continuous vs Rd18	0.36 (0.21–0.62)	0.45 (0.34–0.60)	0.70 (0.58–0.85)	1.10 (0.77–1.57)

Abbreviations: CI, confidence interval; CR, complete response; HR, hazard ratio; MPT, melphalan, prednisone and thalidomide; PR, partial response; SD, stable disease; VGPR, very good partial response.

**Table 3:** Grade 3/4 TEAEs of interest by response groups

*TEAEs*, n *(%)*	*CR (*n=*290)*			⩾*VGPR (*n=*679)*			⩾*PR (*n=*1225)*			⩽*SD (*n=*299)*		
	*Rd Cont (*n=*114)*	*Rd18 (*n=*110)*	*MPT (*n=*66)*	*Rd Cont (*n=*258)*	*Rd18 (*n=*255)*	*MPT (*n=*166)*	*Rd Cont (*n=*432)*	*Rd18 (*n=*425)*	*MPT (*n=*368)*	*Rd Cont (*n=*76)*	*Rd18 (*n=*89)*	*MPT (*n=*134)*
*Hematologic*												
Neutropenia	25 (22)	17 (15)	30 (45)	63 (24)	42 (16)	76 (46)	123 (28)	101 (24)	175 (48)	25 (33)	39 (44)	62 (46)
Anemia	9 (8)	10 (9)	12 (18)	40 (16)	27 (11)	31 (19)	76 (18)	54 (13)	64 (17)	16 (21)	26 (29)	30 (22)
Thrombocytopenia	5 (4)	10 (9)	11 (17)	18 (7)	15 (6)	22 (13)	34 (8)	25 (6)	38 (10)	7 (9)	14 (16)	19 (14)
												
*Nonhematologic*												
Infections	33 (29)	18 (16)	8 (12)	84 (33)	49 (19)	22 (13)	132 (31)	84 (20)	56 (15)	16 (21)	24 (27)	25 (19)
Cardiac disorders	15 (13)	5 (5)	3 (5)	31 (12)	19 (7)	13 (8)	55 (13)	29 (7)	31 (8)	4 (5)	8 (9)	12 (9)
Cataract	13 (11)	4 (4)	1 (2)	27 (10)	9 (4)	2 (1)	32 (7)	12 (3)	3 (1)	1 (1)	2 (2)	0
Deep vein thrombosis	6 (5)	6 (5)	0	18 (7)	11 (4)	4 (2)	25 (6)	18 (4)	11 (3)	4 (5)	2 (2)	3 (2)
Peripheral sensory neuropathy	2 (2)	0	10 (15)	5 (2)	1 (<1)	24 (14)	6 (1)	2 (<1)	42 (11)	0	0	9 (7)

Abbreviations: Cont, continuous; CR, complete response; MPT, melphalan, prednisone and thalidomide; PR, partial response; SD, stable disease; TEAE, treatment-emergent adverse event; VGPR, very good partial response.
