# Supplementary material for: Benefit of continuous treatment for responders with newly diagnosed multiple myeloma in the randomized FIRST trial
Source: Leukemia. 2017 Apr 28;31(11):2435–42. doi: 10.1038/leu.2017.111 (PMC5668494; doi:10.1038/leu.2017.111)
Supplement: Supplementary Table and Figures [file leu2017111x1.docx]

**SUPPLEMENTARY INFORMATION**

Supplementary information includes one table and three figures as cited in the text. All four are included in this Word document.

| Median TTR (range), mo^a^ | CR  (n = 289) | ≥ VGPR  (n = 678) | ≥ PR  (n = 1 223) |
| --- | --- | --- | --- |
| Rd continuous | 1.0  (0.7-4.7) | 1.1  (0.5-8.6) | 1.8  (0.5-22.2) |
| Rd18 | 1.0  (0.8-34.8) | 1.0  (0.8-34.8) | 1.8  (0.8-34.8) |
| MPT | 1.5  (1.4-9.9) | 1.6  (1.3-26.8) | 2.8  (1.2-49.7) |

^a^ Patients with response date before randomization date due to data issue were not included.

**Supplementary Table 1. Time to response by response subgroups.** Median time to first response (TTR) for each response subgroup by treatment arm.

**
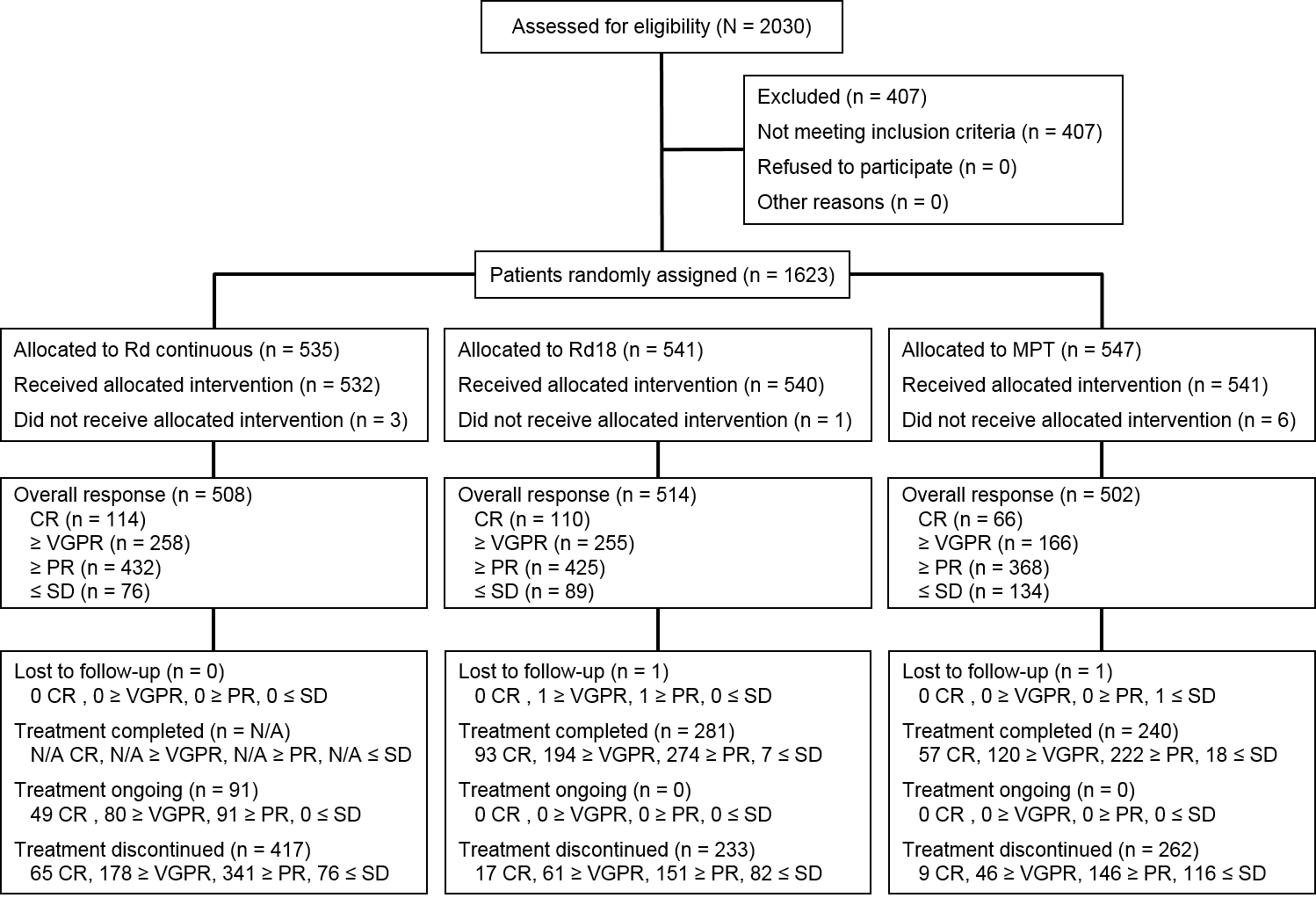
**

**Supplementary Figure 1. Patient flow diagram by depth of response.** CONSORT diagram showing patient disposition. Patients with responses not evaluable were not included in the overall response analysis: Rd continuous, n = 27; Rd18, n = 27; MPT, n = 45.

**
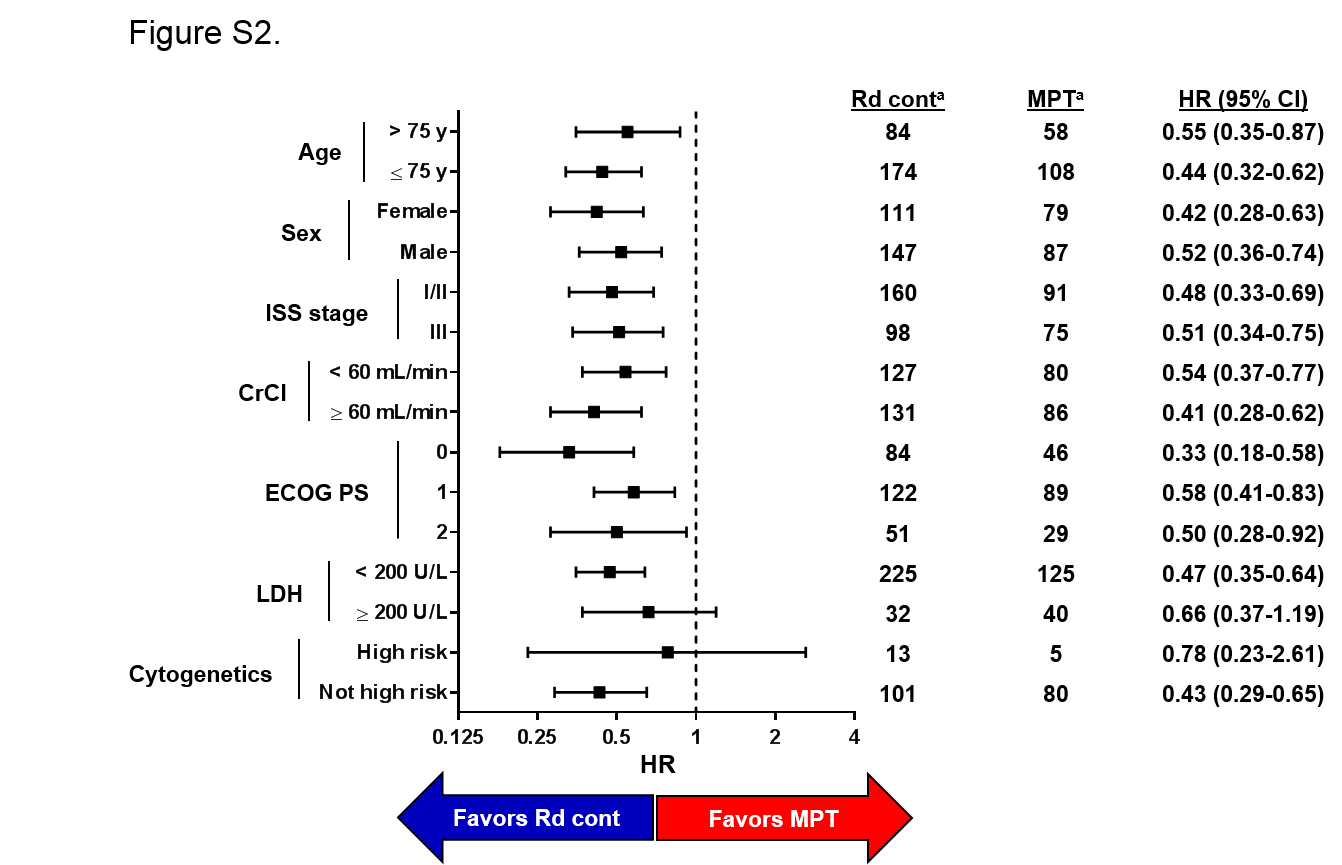
**

**Supplementary Figure 2. Subgroup analyses for duration of response in patients with ≥ VGPR.** Hazard ratios for duration of response in patients with ≥ VGPR by subgroup. ^a^ Number of patients. CrCl, creatinine clearance; ECOG PS, Eastern Cooperative Oncology Group Performance Status; ISS, International Staging System; LDH, lactate dehydrogenase.


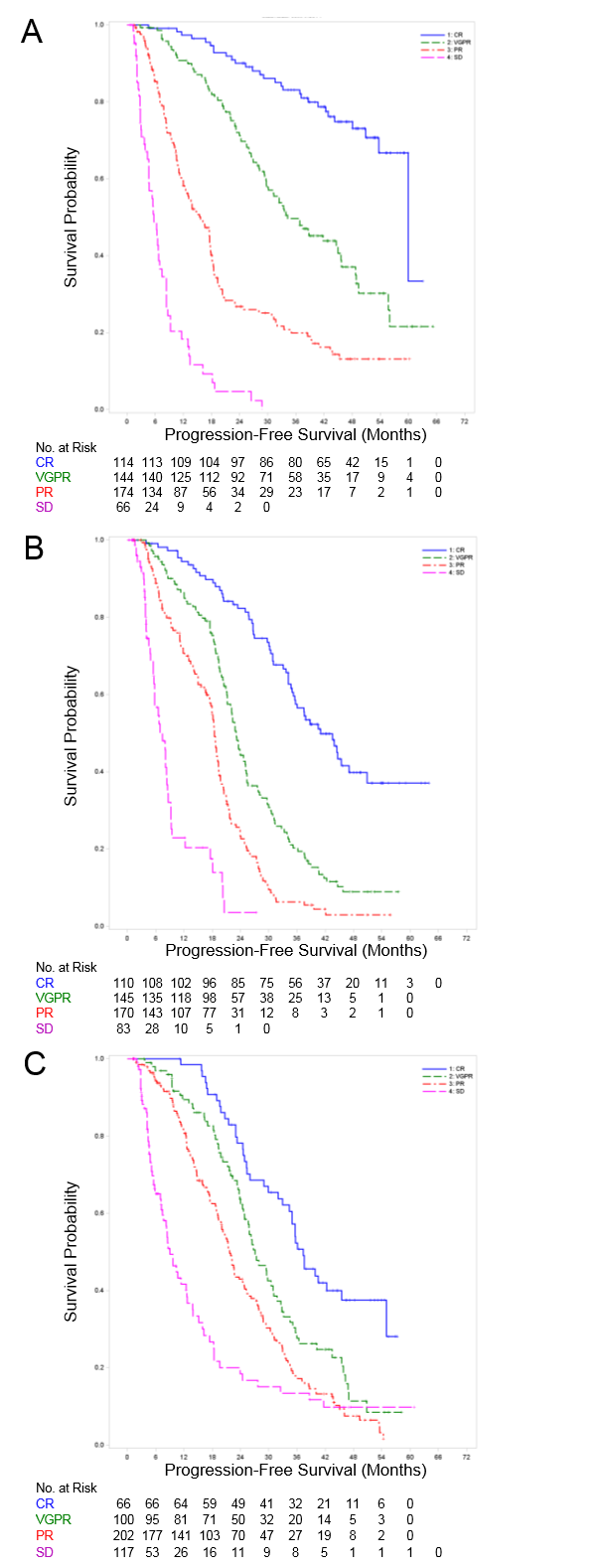


**Supplementary Figure 3. Kaplan-Meier estimates of progression-free survival.** Kaplan-Meier time-to-event curves of progression-free survival for the (A) Rd continuous arm, (B) Rd18 arm, and (C) MPT arm by response subgroups.
